# A Live-Attenuated Chimeric Vaccine Candidate Against the Emerging NADC34-Like PRRSV

**DOI:** 10.3390/vetsci12030290

**Published:** 2025-03-19

**Authors:** Zhengqin Ye, Zhendong Zhang, Zhenbang Zhu, Zhe Sun, Kegong Tian, Xiangdong Li

**Affiliations:** 1 Jiangsu Co-Innovation Center for Prevention and Control of Important Animal Infectious Diseases and Zoonoses, College of Veterinary Medicine, Yangzhou University, Yangzhou 225012, China; 2 National Research Center for Veterinary Medicine, Luoyang 471003, China; 3 Joint International Research Laboratory of Agriculture and Agri-Product Safety, The Ministry of Education of China, Yangzhou University, Yangzhou 225012, China

**Keywords:** PRRSV, NADC34-like strain, vaccine, genomic modification, Marc-145 cell, pigs

## Abstract

NADC34-like porcine reproductive and respiratory syndrome virus (PRRSV) has been circulating in China for several years, causing substantial economic losses to the local pig industry. Current commercial vaccines have failed to provide complete protection against NADC34-like PRRSV infection. Additionally, the poor adaptation of NADC34-like strains to Marc-145 cells presents a considerable challenge for developing effective vaccines against these strains. This study addresses these challenges by developing a novel vaccine candidate against NADC34-like PRRSV. We engineered a recombinant PRRSV, rNADC34-CHSps, by replacing the structural protein region of the JS2021NADC34 strain with that of the CHR6 strain to improve its adaptation to Marc-145 cells. The rescued strain could proliferate well in Marc-145 cells, maintaining high titers and stable growth kinetics even at high passage numbers. Piglets were vaccinated with rNADC34-CHSps at passage 80 and then challenged with the virulent NADC34-like PRRSV strain, JS2021NADC34, at 28 days post-vaccination. All vaccinated piglets developed specific antibodies against PRRSV at 14 dpv and showed no significant clinical symptoms, even after exposure to PRRSV JS2021NADC34. Furthermore, the vaccinated piglets gained significantly more weight, displayed much less severe pathological lesions, and reduced viremia compared to the challenge control piglets. These results indicate that rNADC34-CHSps is a promising vaccine candidate against NADC34-like PRRSV infection, highlighting the potential of targeted genomic modifications to enhance vaccine efficacy.

## Simple Summary

The porcine reproductive and respiratory syndrome virus (PRRSV) poses a significant challenge to swine production, leading to substantial economic losses. NADC34-like strains with varying pathogenicity have circulated in China for several years due to the limited effectiveness of commercial vaccines. This study aims to develop a live attenuated vaccine candidate against NADC34-like PRRSV. A recombinant virus, rNADC34-CHSps, was constructed, which exhibited stable replication in Marc-145 cells and demonstrated safety in pigs. Furthermore, vaccination with rNADC34-CHSps induced significant protection against challenges with a virulent NADC34-like strain. This study provides a promising vaccine candidate for controlling NADC34-like PRRSV infections, highlighting the potential of targeted genetic manipulation to enhance vaccine efficacy.

## 1. Introduction

Porcine reproductive and respiratory syndrome (PRRS) is a globally significant infectious disease of swine caused by the porcine reproductive and respiratory syndrome virus (PRRSV) [1]. PRRSV is an enveloped virus belonging to the order *Nidovirales*, family *Arteriviridae*, characterized by a positive-sense, single-stranded RNA genome of approximately 15 kb [2,3]. The PRRSV replicase gene, encoded by ORFs 1a and 1b, generates two polyproteins, subsequently cleaved into at least 14 non-structural proteins (Nsps) by four ORF1a proteases (Nsp1α, Nsp1β, Nsp2, and Nsp4). Furthermore, ORF2-7 encodes four glycoproteins (GP2a, GP3, GP4, and GP5), three unglycosylated proteins (E, ORF5a, and M), and a nucleocapsid protein (N) [4]. Crucially, non-structural proteins are essential for viral replication, while structural proteins contribute to virus assembly and entry [4].

Since its initial discovery, PRRSV has consistently evolved and is typically classified into two main genotypes, PRRSV-1 and PRRSV-2, according to genetic diversity. Both genotypes exhibit significant genetic variability, leading to the emergence of new, virulent strains that cause outbreaks worldwide. The Nsp2 protein of PRRSV exhibits significant variation across different lineages, characterized by deletions of varying lengths and locations, including a 12-aa deletion in HB-2(sh)/2002 [5], a 30-aa discontinuous deletion in HP-PRRSV [6], a 131-aa discontinuous deletion in NADC30 strains [7], and a unique 100-aa continuous deletion in NADC34-like strains [8]. The emergence of the PRRSV-2 NADC34 strain in the United States was firstly reported in 2012 and characterized by severe reproductive losses [9]. Researchers have observed a notable rise in the pathogenicity of NADC34-like PRRSV, with the IA/2014/NADC34 prototype demonstrating high pathogenicity to piglets [10]. In China, PRRSV variants display a range of virulence, from mild (e.g., HLJDZD32-1901, a NADC34-like PRRSV) to moderate (e.g., PRRSV-ZDXYL-China, an ORF5 RFLP 1-7-4 PRRSV) and high (e.g., JS2021NADC34, a NADC34-like PRRSV) [11,12,13]. NADC34-like PRRSV has become the predominant strain in some areas of China. Our previous work [14], along with other studies [15], has shown that commercial PRRSV vaccines provide incomplete protection against highly pathogenic NADC34-like strains, including JS2021NADC34 and PRRSV/CN/FJGD01/2021. Therefore, it is urgent to develop effective vaccines against NADC34 PRRSV infection.

So far, the most effective method to prevent PRRS remains to be vaccination. Various vaccines are currently available or under development, including inactivated vaccines, modified live virus (MLV) vaccines, vector vaccines, subunit vaccines, DNA vaccines, and RNA vaccines [16]. MLV vaccines have been used for over 30 years to combat PRRSV infections, which could elicit a protective immune response that mimics natural infection [16,17,18]. Nevertheless, the continuous evolution of PRRSV, particularly the emergence of highly pathogenic strains, has posed a challenge to developing next-generation MLV vaccines. Overcoming this challenge requires a comprehensive understanding of the viral factors that elicit a robust and protective immune response. Specifically, identifying the key viral regions capable of inducing neutralizing antibodies is crucial for rational vaccine design. Many researchers assert that the neutralization region of PRRSV is located within ORF2-6, leading to the development of numerous genetically engineered vaccines targeting this area [18,19]. Importantly, Chaoliang Leng et al. demonstrated that the ORF1a region of PRRSV also functions as a neutralization region by generating and testing chimeric clones derived from an attenuated HP-PRRSV and a classic PRRSV strain [20,21].

PRRSV exhibits a restricted cellular and host tropism, with porcine alveolar macrophages (PAMs) and blood monocytes as primary target cells [22]. Several African green monkey-derived cell lines, including MA104, CL2621, and Marc-145, are permissive to PRRSV infection and have been extensively used for PRRSV isolation and vaccine development [23]. However, the continuous evolution of PRRSV, coupled with increasing strain diversity, limits the replication of certain strains, including NADC30-like, QYYZ-like, NADC34-like, and highly pathogenic (HP)-PRRSV variants, in Marc-145 cells [7,24]. Consequently, the traditional method [25,26,27,28,29] of serial passaging in Marc-145 cells to create attenuated vaccine strains is not applicable for developing vaccines for these poorly replicating PRRSV variants. Recent studies have demonstrated that PRRSV variations in the structural proteins, particularly the minor envelope protein complex (specifically GP2a and GP3), are key determinants of PRRSV tropism for Marc-145 cells, thus providing a clue for vaccine development targeting PRRSV strains that cannot efficiently infect Marc-145 cells [24,29].

This study details the development of an attenuated, recombinant NADC34-like PRRSV. The recombinant was generated through targeted substitution of the genomic segment encoding the structural proteins of the JS2021NADC34 strain with the corresponding sequence from the CHR6 strain, a CH-1R-like vaccine strain, exhibiting robust replication in Marc-145 cells. This modified virus demonstrates a strong capacity for replication in Marc-145 cells and provides significant protection against the challenge of the highly pathogenic JS2021NADC34 strain.

## 2. Materials and Methods

### 2.1. Cells and Viruses

HEK 293T cells were used for the recovery of the virus from in vitro DNA transfection. Marc-145 cells were employed for virus rescue, culture, and subsequent experiments. The cells were maintained at 37 °C in a 5%CO_2_ atmosphere using Dulbecco’s Modified Eagle Medium (DMEM) supplemented with 10% fetal bovine serum (FBS), as previously described [30]. CHR6, a CH-1R-like PRRSV strain, was previously reported [31,32]. JS2021NADC34 PRRSV (GenBank: MZ820388.1), a NADC34-like PRRSV strain, was isolated and characterized in our laboratory following previously established protocols [13].

### 2.2. Construction of Plasmid, Transfection, and Passage

**Figure 1:**
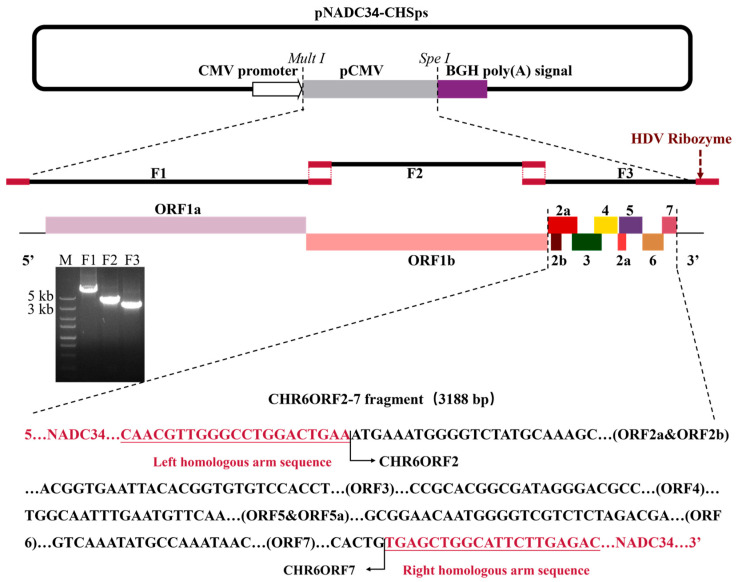
Strategy to construct the full-length cDNA clone of the PRRSV rNADC34-CHSps strain. The strategy was modified from previous studies [33]. The pCMV plasmid, including the unique restriction enzymes, is shown in the top part. The first two overlapped fragments of the JS2021NADC34 genome and the third overlapped fragment of the CHR6 genome were generated by PCR amplification and are displayed in the middle part. The first two fragments were shown in a previous study [33]. The third fragment is shown in the bottom part.

The pCMV-JS2021NADC34 plasmid, previously described in our lab [33], was used as a backbone. This plasmid was divided into three overlapping fragments (F1, F2, and F-Vec), excluding the ORF2-7 region. The coding sequence of the CHR6 ORF2-7 was then amplified from the complete genomic cDNA of the CHR6 strain, generating the F3 fragment. These four fragments were subsequently combined through homologous recombination technology [33]. The whole strategy was divided into two steps (Figure 1). The cDNA of the CHR6 strain was obtained via the previously described method [33]. The cDNA of the CHR6 strain and the pCMV-JS2021NADC34 plasmid served as templates and were amplified using Phanta Max Super-Fidelity DNA Polymerase (Vazyme, Nanjing, China), according to the manufacturer’s protocol. Primers for the four amplified fragments are listed in Table 1 (underlined sequences indicate homology arms). The four fragments were assembled into a full-length cDNA clone, designated as pNADC34-CHSps. The infectious cDNA clone plasmid was isolated using an EndoFree Plasmid Mini Kit (CW Bio, Taizhou, China), adhering to the manufacturer’s protocol. HEK-293T cells, at approximately 90% confluency in twelve-well plates, were transfected with 1 μg of purified plasmid DNA and 3 μL PEI reagent (YEASEN, Shanghai, China), following the manufacturer’s protocol. The supernatant was collected, labeled as PRRSV rNADC34-CHSps, and was then used to inoculate Marc-145 cells.

### 2.3. Characterization of rNADC34-CHSps

To verify the adaptation of the rNADC34-CHSps virus to Marc-145 cells, the rNADC34-CHSps virus was inoculated into Marc-145 cells at a multiplicity of infection (MOI) of 1, with the parental strains JS2021NADC34 and CHR6 serving as controls. Immunofluorescence assay (IFA) and Western blotting (WB) were employed to detect the PRRSV-2 nucleocapsid protein at 48 h post-infection (hpi), as previously described [33,34].

To assess the genetic composition of the recombinant virus, RNA was extracted from the passage 5 (P5) virus using a Viral RNA Mini kit (TransGen Biotech, Beijing, China), following the manufacturer’s instructions. The cDNA was obtained via the previously described method [33]. To prevent carryover DNA contamination from transfections during passage, the passage 5 (P5) virus RNA preparations were treated with gDNA Wiper (Vazyme, Nanjing, China). Four fragments were then amplified using the primers indicated in Table 1. Four overlapping fragments were then amplified by PCR using the primer pairs listed in Table 1: fragment 1A (JS2021NADC34F1-LF/JS2021NADC34F1A-R), fragment 1B (JS2021NADC34F1B-F/JS2021NADC34F1-LR), fragment 2 (JS2021NADC34F2-LF/JS2021NADC34F2-LR), and fragment 3 (CHR6F3-LF/CHR6F3-LR). Amplicons were purified using a DNA mini-purification kit (UElandy, Suzhou, China) and then ligated into a pMD19-T vector using a TA cloning kit (Takara, Osaka, Japan) prior to being sequenced.

### 2.4. Characterization of In Vitro Growth Properties in Marc-145 Cells

Marc-145 cells were infected with both CHR6 virus and rNADC34-CHSps recombinant virus at a multiplicity of infection (MOI) of 1. Supernatants from the infected cell cultures were collected at 0, 12, 24, 36, 48, 60, and 72 hpi, and viral titers were determined by TCID_50_ assays [30].

### 2.5. Animal Experiment

Ten four-week-old, PRRSV-free, large White–Duroc crossbred pigs were randomly divided into two groups (*n* = 5 per group). One group received an attenuated rNADC34-CHSps virus vaccination (2 × 10^6^ TCID_50_, passage 80) via combined intranasal (0.5 mL/nostril) and intramuscular (0.5 mL) routes [35], while another group served as the unvaccinated control, which received DMEM medium as a placebo. At 28 days post-vaccination (dpv), all pigs were challenged with JS2021NADC34 PRRSV (5 × 10⁵ TCID₅₀/pig) via combined intranasal (0.5 mL/nostril) and intramuscular (0.5 mL) routes, following the established protocols [35]. Pigs were monitored daily for rectal temperature and clinical signs [35], and their clinical condition was assessed using a numerical index reflecting disease severity [26]. Lung pathology was evaluated by calculating the percentage of lesions per lobe and determining an overall gross pathology score using a standard system [14]. Blood samples were collected from the anterior vena cava into 1.5 mL centrifuged tubes using 0.6 mm × 25 mm needles (TWLB). The collected blood was incubated at 37 °C for 30 min and then centrifuged at 12,000 r/min for 5 min to separate the serum. The serum was then stored at −20 °C for subsequent viremia and antibody detection. Pigs were humanely euthanized by pentobarbital sodium (intravenous injection, 150 mg/kg) following the established procedures [14] when they reached moribund conditions or at the study endpoint (14 days post-challenge (dpc)). Lung, lymph node, and tonsil samples were removed and analyzed for viral RNA using Taq-Man^®^-based real-time RT-PCR and for pathological changes using H&E staining and immunohistochemistry [33]. Body weight gain, survival, and mortality were recorded and calculated [14]. The piglets were housed in the Experimental Animal Center of Yangzhou University, and the animal experimental protocol was approved by the Animal Welfare and Ethics Committee of Yangzhou University (reference number 202411008), and all procedures were performed according to conventional animal welfare guidelines.

### 2.6. Viremia and Serological Test

Serum samples from vaccinated and unvaccinated pigs were collected at 0, 7, 14, 21, and 28 dpv and at 7 and 14 dpc. Viral RNA was quantified by qPCR, following the established protocols, and primers and probes used for viral quantification were listed below: PRRSV2-UF: TTGTGCTTGCTAGGCCGC; PRRSV2-UR: ACGACAAATGCGTGGTTATCA; PRRSV-UP: FAM-TCTGGCCCCTGCCCA-MGB [36]. PRRSV-specific antibody levels were measured using a JNT ELISA kit (JNT, Beijing, China) according to the manufacturer’s instructions and expressed as sample-to-positive (S/P) ratios. Samples with an S/P ratio <0.4 were considered negative, while those ≥0.4 were considered positive. PRRSV-neutralizing antibody titers in serum were determined by the FFN assay, following the established procedures [37]. A titer ≥1:4 was considered positive. All assays were performed in triplicate.

### 2.7. Histopathology and Immunohistochemistry Staining

Lung, lymph node, and tonsil samples collected at necropsy were processed for histopathology and immunohistochemistry (IHC) staining according to the established protocols [13,14]. Automated staining was performed using a Leica fully automated staining machine. An anti-PRRSV N antibody (4A5, MEDIAN, Republic of Korea) was used for IHC staining.

### 2.8. Statistical Analyses

Statistical analysis was performed using GraphPad Prism 9 software. Data are presented as mean ± standard deviation (SD). Two group comparisons were conducted using an unpaired Student’s *t*-test with unequal variances. *p* < 0.05 was considered to be significant.

## 3. Results

### 3.1. Generation of rNADC34-CHSps Virus

**Figure 2:**
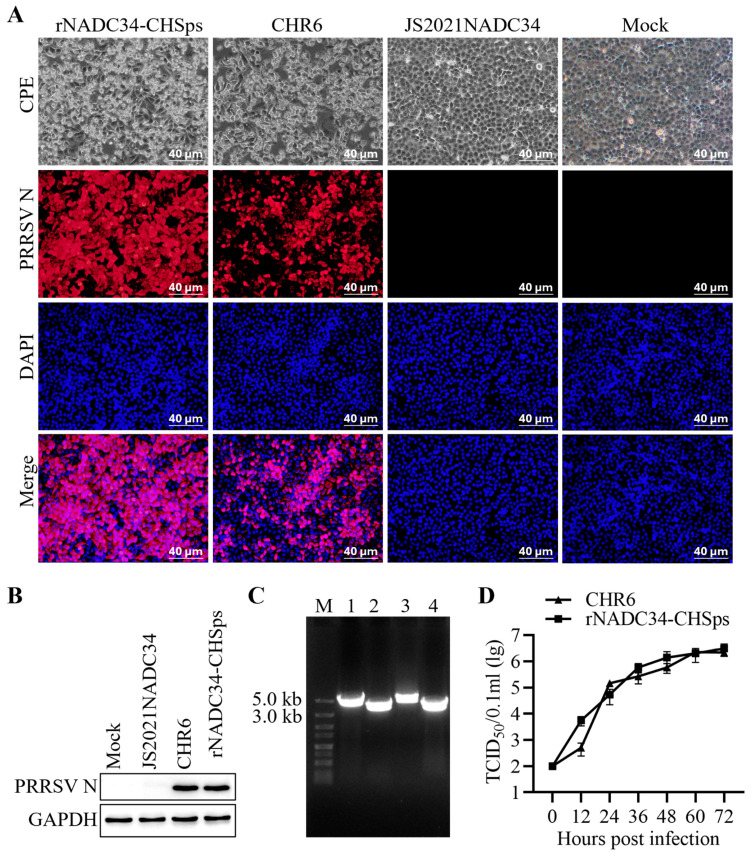
Identification of the rescued rNADC34-CHSps virus. Marc-145 cells were either mock-infected or infected with the parental viruses (CHR6 and JS2021NADC34) and rNADC34-CHR6-Sps virus at a multiplicity of infection (MOI) of 1. (**A**) CPE and IFA target the PRRSV N protein in Marc-145 cells. CPE was observed at 72 hpi. While cells were stained with a monoclonal antibody (mAb) recognizing the viral nucleocapsid protein (red), and nuclei were counterstained with DAPI (blue) at 48 hpi. The cells infected or mock-infected with CHR6 or JS2021NADC34 strains are shown as controls. Scale bar = 40 µm. (**B**) Western blot analysis of viral N protein levels in the infected or mock-infected Marc-145 cells at 48 hpi. GAPDH was shown as an internal control. Cells mock-infected or infected with CHR6 or JS2021NADC34 strains served as controls. (**C**) RT-PCR analysis of the complete rNADC34-CHR6-Sps genome. Amplification of the complete rNADC34-CHR6-Sps viral genome was achieved using four overlapping PCR products. Lane M: DNA marker DL5000; Lanes 1–4: Four overlapping PCR products of the viral genome. (**D**) Growth kinetics of rNADC34-CHSps in Marc-145 cells. Cells were infected at an MOI of 1, and viral titers in the supernatants were determined using TCID_50_ assays at the indicated time points (0, 12, 24, 36, 48, 60, and 72 hpi). Cells infected with CHR6 served as a control. Data are presented as mean ± SD (n = 3).

The JS2021NADC34 strain served as the backbone for creating a chimeric viral mutant. The genome of the cloned rNADC34-CHSps virus, containing the structural proteins (SPs) region from the CHR6 strain, was determined to be 15,132 bp, excluding the poly(A) tail. As illustrated in Figure 1, the full-length cDNA clone of the rNADC34-CHSps virus includes a CMV immediate-early eukaryotic promoter at the 5′ end, a 15,132 bp genome, a 22 nt poly(A) tail at the 3′ end, followed by an HDV ribozyme and BGH poly(A) signal sequences. The plasmid was directly used for DNA transfection into HEK-293T cells. Supernatants collected from the transfected cells at 48 hpi were then passaged in Marc-145 cells. Cytopathic effects (CPE) became evident by 3 days post-infection (dpi). IFA and WB analysis demonstrated that the infectious clone could replicate in Marc-145 cells. Mock-infected cells, alongside cells infected with the parental JS2021NADC34 and CHR6 viruses, served as controls (Figure 2A,B; original WBs are available in the Supplementary Material).

To ascertain the recombinant origin of the rescued virus (rNADC34-CHSps), RNA from passage 5 was subjected to further analysis. RT-PCR products (Figure 2C) obtained from rNADC34-CHSps cDNA were sequenced, confirming that the rescued virus retained the precise nucleotide sequence of the original plasmid pNADC34-CHSps. In vitro growth kinetics assays using rNADC34-CHSps (P5) and CHR6 on Marc-145 cells demonstrated comparable replication profiles (Figure 2D).

### 3.2. Growth Kinetics of Different Passages of rNADC34-CHSps Virus

**Figure 3:**
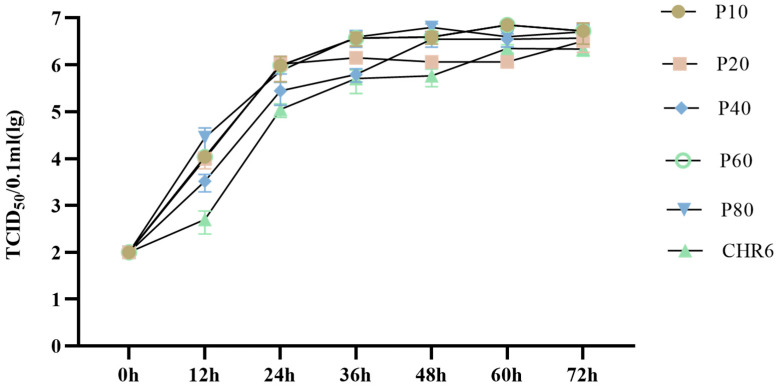
In vitro replication kinetics of rNADC34-CHSps virus at different passages. Marc-145 cells were infected with rNADC34-CHSps or CHR6 virus at an MOI of 1. Viral titers in supernatants were determined by TCID_50_ assays at the indicated time points (0, 12, 24, 36, 48, 60, and 72 hpi). Data are shown as mean ± SD from three independent experiments.

To develop a live-attenuated NADC34 PRRSV vaccine, the rNADC34-CHSps virus was serially passaged in Marc-145 cells, up to 80 passages. Growth was assessed at passages 10, 20, 40, 60, and 80. No significant difference in growth rates was observed at higher passages compared with passage 10 and the CHR6 strain (Figure 3).

### 3.3. Clinical Performance After Immunization and Challenge

**Figure 4:**
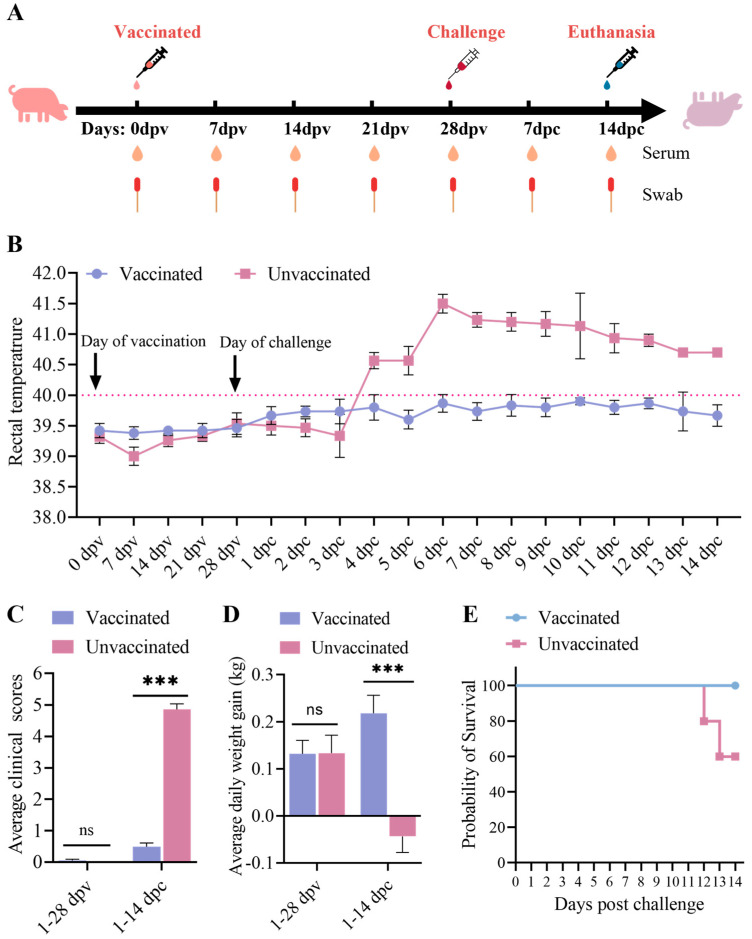
Clinical performance after vaccination and challenge. (**A**) Overview of the animal experiment protocol. Body temperature (**B**), clinical signs (**C**), daily body weight gain (**D**), and survival rate (**E**) of pigs in two groups. Fever was set as above 40.0 °C. The body weights of pigs were measured at 28 dpv and 14 dpc. *** Indicates a statistically significant difference (*p* < 0.001), and ns indicates insignificant.

After vaccination, no clinical signs of PRRS were observed in either the vaccinated or unvaccinated piglets. After the JS2021NADC34 PRRSV challenge, the vaccinated piglets showed no significant changes in body temperature, clinical signs, or weight loss (Figure 4B–D). In contrast, the unvaccinated piglets displayed various disease manifestations starting at 4 dpc, including a prolonged fever (up to 40.5 °C persisting for 4–14 days) (Figure 4B). The unvaccinated piglets also showed variable degrees of cough, white purulent nasal discharge, anorexia, and emaciation. Compared to the vaccinated group, unvaccinated piglets demonstrated significantly less body weight gain (*p* < 0.05) at 1–14 dpc (Figure 4D). One of five unvaccinated pigs was euthanized due to its moribund conditions at 12 dpc, and another unvaccinated pig died at 13 dpc (Figure 4E). The remaining pigs were euthanized at 14 dpc.

### 3.4. Antibody Responses of the Piglets

**Figure 5:**
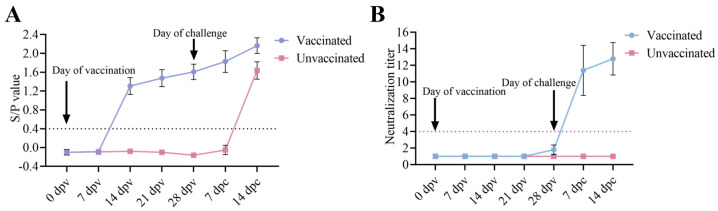
Dynamics of antibody generation in two groups. (**A**) Antibody levels of PRRSV N protein in pigs were measured in two groups. The dashed line indicates the threshold value above ≥0.4, in which titers were considered positive for anti-PRRSV antibodies. (**B**) Neutralizing antibody titers against the JS2021NADC34 strain in vaccinated and unvaccinated pigs. The dashed line indicates the threshold for positivity (titer ≥ 1:4).

ELISA results indicated that all vaccinated piglets had seroconverted by 14 dpv (Figure 5A). As expected, PRRSV-specific antibodies were not detected in unvaccinated piglets prior to the challenge (Figure 5A). After the JS2021NADC34 PRRSV challenge, ELISA titers of the vaccinated pigs kept increasing, and the unvaccinated pigs had seroconverted by 14 dpc. The neutralizing antibody titers of the vaccinated pigs seroconverted by 28 dpv and kept increasing until 14 dpc (Figure 5B). There was no detectable neutralizing antibody for the unvaccinated pigs.

### 3.5. Viremia, Virus Shedding, and Virus Tissue Distribution Between the Immunized-Challenge Group and the Challenge Group

**Figure 6:**
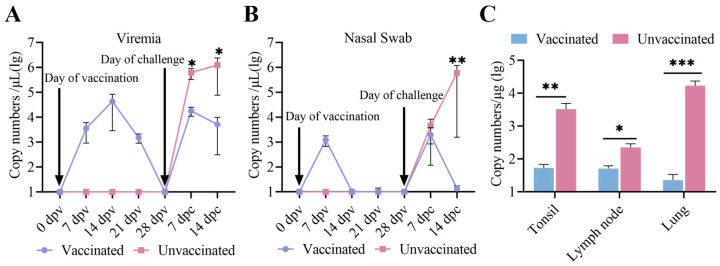
Viral load and distribution in two groups. Viremia (**A**), viral loads in nasal swab (**B**), and viral loads in three tissues (**C**) of pigs in two groups. *, **, and *** indicate a statistically significant difference (*p* < 0.05, 0.01, 0.001), respectively.

To further assess differences in viremia, viral shedding, and tissue distribution between the two groups, serum samples and nasal swabs collected at 0, 7, 14, 21, and 28 dpv and 7 and 14 dpc, along with three organ tissues, were evaluated using real-time PCR [36]. As demonstrated in Figure 6A, viral RNA copy numbers in the serum of vaccinated piglets peaked at 14 dpv, subsequently declined, and were undetectable prior to the challenge. Following the challenge, serum viral RNA copy numbers in vaccinated piglets peaked again at 7 dpc and then decreased. By contrast, serum viral RNA copy numbers in unvaccinated piglets kept increasing after viral challenge. The viremia levels in vaccinated piglets were significantly lower than those in unvaccinated piglets throughout the viral challenge period. Live viral shedding, as determined by qRT-PCR analysis, was detectable only at 7 dpc in the vaccinated piglets, whereas viral shedding in the unvaccinated piglets showed a continuing upward trend (Figure 6B). Viral loads in all three tissues in the vaccinated piglets were significantly lower compared to the unvaccinated piglets (Figure 6C).

### 3.6. Gross Pathological and Histopathological Changes

**Figure 7:**
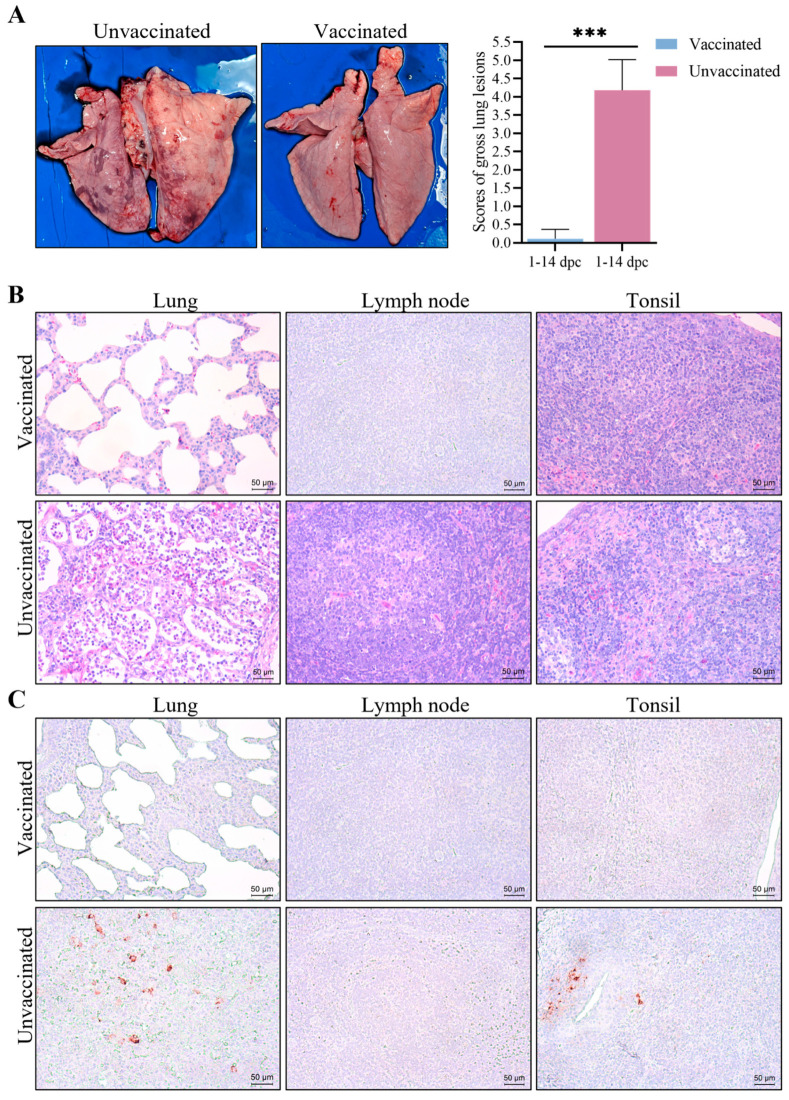
Gross pathological, histopathological changes, and IHC stains in two groups. (**A**) Lung pathological scores of pigs in two groups. Gross lung lesion scores were calculated and averaged. *** Indicated a statistically significant difference (*p* < 0.001). (**B**) Histopathological analysis of lung, lymph node, and tonsil samples from the vaccinated and the unvaccinated piglets. Original magnification, ×200; scale bar = 50 µm. (**C**) IHC staining of lung, lymph node, and tonsil samples from the vaccinated and unvaccinated piglets. PRRSV antigens are indicated by brown staining using a PRRSV-specific antibody. Original magnification, ×200.

At necropsy, the unvaccinated piglets displayed severe lung lesions (Figure 7A). The histopathological analysis further indicated substantial inflammatory cell infiltration, epithelial cell proliferation, and marked alveolar septal thickening in the lungs of unvaccinated piglets (Figure 7B). Additionally, lymph nodes from the unvaccinated piglets showed lymphocyte depletion and medullary hemorrhage (Figure 7B). In contrast, the vaccinated piglets exhibited minimal lung pathology and only mild lymphocyte depletion in lymph nodes (Figure 7B). Tonsils from the unvaccinated pigs had acute hemorrhage, lymphocyte depletion, and infiltration of neutrophils. IHC staining, using a PRRSV-specific monoclonal antibody, showed positive cells in the lungs and tonsils of the unvaccinated piglets, whereas no positive cells were observed in the vaccinated piglets (Figure 7C).

## 4. Discussion

PRRS has remained a significant global threat to the swine industry since the late 1980s. Surveillance shows a clear increase ratio of NADC34-like PRRSVs in the field since 2021 [38]. The isolation of the highly pathogenic TJnh2021 strain in Tianjin in 2021, a natural recombinant of IA/2014/NADC34 and QYYZ [39], highlighted the diverse virulence of NADC34-like PRRSV, contrasting with the moderate-to-low virulence of PRRSV-ZDXYL-China-2018-1 [39,40]. Our previous research demonstrated the significant pathogenicity of the JS2021NADC34 strain in nursery pigs, resulting in high mortality rates [13]. Importantly, existing commercial PRRSV vaccines often fail to provide complete protection against highly pathogenic NADC34-like strains [14,15]. This limited efficacy highlights a critical gap in control strategies and emphasizes the urgency for improved vaccines tailored to address these emerging viral variants.

Since the 1990s, research efforts focused on PRRSV vaccines have progressed from live virus immunization (LVI) to the development and commercialization of PRRSV-MLV, incorporating strategies that include immune downregulation, marker and multivalent vaccines, self-adjuvanted vaccines, and chimeric viruses [16]. While LVI has proven effective in eradicating PRRSV, it carries many risks, including virus shedding and the potential for contamination. To secure safer vaccines, the first MLV was introduced in the U.S. in 1994, leading to considerable research into its safety and efficacy [16]. Commercially available PRRSV-MLV vaccines have been produced through repetitive passage in cell culture under various proprietary selective pressures [23]. The majority of cell lines utilized were sourced from the MA-104 monkey kidney cell line, which is the sole widely accessible continuous cell line that facilitates the replication of PRRSV [23]. Although PRRSV-MLV vaccination frequently shows incomplete cross-protection, numerous studies have indicated that it provides protective efficacy against wild-type isolates from the US, EU, and Asia by reducing clinical signs, alleviating body weight loss, decreasing lung lesions, lowering viral shedding, and enhancing various clinical and production parameters [15]. Recent reverse genetics systems using cDNA clones from PRRSV isolates have advanced live vaccine safety and efficacy. The tools of molecular biology, such as restriction endonucleases, bacterial cloning, PCR, and site-directed mutagenesis, are well suited for the modification of DNA sequences. Kwon B et al. developed a chimeric arterivirus using infectious cDNA clones, substituting genes from one PRRS strain with those from another strain, a different PRRS genotype, or a non-PRRS variant [41]. The recovered chimeric virus exhibited the broad cell tropism of EAV, unlike the narrow tropism of PRRSV [22]. Zhao K et al. utilized an infectious cDNA clone derived from an attenuated Chinese PRRSV alongside one from the Chinese HP-PRRSV to generate chimeric viruses with modified virulence successfully [42]. Infectious clones of the attenuated vaccine strain Ingelvac^®^MLV from VR2332 and the virulent Lineage 1 field virus from MN184 were utilized to create several chimeric viruses. These chimeras exhibited diverse phenotypes concerning replication, virulence, and protective efficacy [26,43].

NADC34-like PRRSV strains exhibit poor adaptation to Marc-145 cells, presenting a challenge for the development of attenuated vaccines against these specific strains [13,33]. Zhang et al. have confirmed that the significant variations in non-Marc-145-adaptive PRRSVs are in the ORF2-7 gene regions, especially in ORF2-4 [24]. In this study, we constructed a recombinant PRRSV rNADC34-CHSps containing the structural protein-encoding region from the CHR6 strain (a CH-1R-like PRRSV strain) and demonstrated its successful replication in Marc-145 cells through IFA and WB (Figure 2A,B). Subsequently, we passaged rNADC34-CHSps in Marc-145 cells up to 80 passages to develop an attenuated vaccine. Notably, the titers were consistently maintained between 10^6^ and 10^7^ TCID_50_/0.1 mL, and the growth kinetics showed no significant differences from the parental strain CHR6 in Marc-145 cells, even after numerous passages, indicating its efficacy for propagation in Marc-145 cells (Figure 3). Importantly, pigs vaccinated with a high dose (2 × 10^6^ TCID_50_) of rNADC34-CHSps at passage 80 stayed healthy, with no clinical symptoms evident before the challenge, suggesting that this strain is safe for pigs (Figure 4B–D).

Effective control of PRRSV infection requires the generation of neutralizing antibodies (Nabs), which are critical for viral clearance. Although PRRSV antibody production is initiated within 7 to 9 dpi, these early antibodies are generally not protective. Typically, Nabs emerge later, approximately 28 days after infection [44]. Many researchers have focused on the neutralization region of PRRSV located within ORF2–6, and genetically modified vaccines targeting this area have been developed [18,19]. However, recent findings by Leng et al. [20] have demonstrated that ORF1a also functions as a key neutralization region for PRRSV. Notably, NADC34-like PRRSV strains, including the JS2021NADC34 strain used in this study, are characterized by variations in the Nsp2 encoded by ORF1a [44,45]. In this study, we observed that rNADC34-CHSps, which contains the ORF1ab of the JS2021NADC34 strain, successfully replicated in pigs and induced robust levels of antibodies against PRRSV N proteins, detected at 14 dpv, with antibody levels continuing to increase throughout the experiment (Figure 5A). Crucially, our study demonstrates that the rNADC34-CHSps elicited Nabs. Neutralizing antibody titers against JS2021NADC34 were low (≤1:2) at 28 days after vaccination. A significant rise to 1:12 occurred within seven days, with titers continuing to increase until the end of the study (Figure 5B). This early and robust Nab response suggests the potential for robust and longer-lasting protection conferred by the rNADC34-CHSps.

To further evaluate the protective effect of rNADC34-CHSps against the JS2021NADC34 strain, pigs were challenged at 28 dpv. The results clearly demonstrated that the rNADC34-CHSps vaccine candidate offered good protection against the PRRSV JS2021 PRRSV infection. Importantly, the vaccinated pigs exhibited no significant clinical symptoms; they displayed reduced fever scores and coughing, and a statistically significant increase in average weight gain compared to the unvaccinated group. Pathological analysis revealed that rNADC34-CHSps effectively prevented the development of gross and microscopic lung lesions in vaccinated piglets. These above findings suggest that our PRRSV vaccine candidate, rNADC34-CHSps, has the potential to offer enhanced protection against NADC34-like PRRSV infection. However, we acknowledge that the viremia observed in vaccinated pigs during this study appeared at 7 dpv, peaked at 14 dpv, then declined quickly and became undetectable by 28 dpv, ranging from 10^3.0^ to 10^4.5^ copy numbers/μL (Figure 6A). Of particular interest, this level of viremia was significantly higher than that reported for the SD-R strain in previous studies [35], a finding that warrants further examination. This is an interesting observation, as some previous research suggests that higher and more sustained viremia in piglets can indicate a greater capacity for PRRSV replication, potentially leading to enhanced antibody production [20]. Conversely, the CH-1R strain, as investigated by Chaoliang Leng et al., failed to elicit any detectable antibody response or viremia in piglets [20]. These conflicting findings underscore the complex relationship between viremia, antibody response, and vaccine efficacy and highlight that the protective mechanism of different vaccines remains unclear. This study found that rNADC34-CHSps induced low neutralizing antibody titers (≤1:2) at 28 dpv, which only increased significantly to 1:12 by 7 dpc. This suggests that the vaccine’s protective mechanism may not rely entirely on neutralizing antibodies but could involve cell-mediated immunity. Beyond understanding immune correlations, future research must also address potential drawbacks such as recombination risks. Specifically, we aim to elucidate the molecular mechanisms underlying the observed attenuation, determine the duration of protection under field conditions, and gather comprehensive long-term safety data. Despite these remaining questions, progress in PRRS vaccinology continues towards the development of a new generation of safer and broadly cross-protective live vaccines.

## 5. Conclusions

This study successfully developed an attenuated recombinant virus, rNADC34-CHSps. It demonstrated efficient replication in Marc-145 cells and provided effective protection against a highly pathogenic NADC34-like PRRSV infection in piglets. These findings suggest that rNADC34-CHSps could be a promising live-attenuated vaccine candidate and merit further investigation.

## Figures and Tables

**Table 1:** Information on primers used in the study.

Primer	Sequence (5′-3′)
JS2021NADC34F1-LF	CAGAGCTGCATGCTAATAGCATGACGTATAGGTGTTGGCTCTATG
JS2021NADC34F1-LR	TAAGGAGCAGTGTTTAAACTGCTAGC
JS2021NADC34F2-LF	GCAGTGTTTAAACTGCTAGCCGCCA
JS2021NADC34F2-LR	TTCAGTCCAGGCCCAACGTTGGTTCAAC
CHR6F3-LF	AACGTTGGGCCTGGACTGAAATGAAATGGGGTCTATGCAA
CHR6F3-LR	GTCTCAAGAATGCCAGCTCATCATGCTGAGGGTGATGC
Vec-F	TGAGCTGGCATTCTTGAGACATCCCAGTGCTTG
Vec-R	GCTATTAGCATGCAGCTCTGCTTATATAG
JS2021NADC34F1B-F	TTATTGGGCGGGGCACGCTACGTCT
JS2021NADC34F1A-R	AGACGTAGCGTGCCCCGCCCAATAA

## Data Availability

All Western blots presented in figures were given in the section of Supplementary Materials, which are named Original Data of WBs. The data that support the findings of this study are available from the corresponding author upon reasonable request.

## Supplementary Materials

The following supporting information can be downloaded at: https://www.mdpi.com/article/10.3390/vetsci12030290/s1, Supplementary File: Original Data of WB blots.

## References

[1] Chaudhari J., Vu H.L.X. (2020). Porcine Reproductive and Respiratory Syndrome Virus Reverse Genetics and the Major Applications. Viruses.

[2] Balasuriya U.B., Carossino M. (2017). Reproductive effects of arteriviruses: Equine arteritis virus and porcine reproductive and respiratory syndrome virus infections. Curr. Opin. Virol..

[3] Fiers J., Cay A.B., Maes D., Tignon M. (2024). A Comprehensive Review on Porcine Reproductive and Respiratory Syndrome Virus with Emphasis on Immunity. Vaccines.

[4] Music N., Gagnon C.A. (2010). The role of porcine reproductive and respiratory syndrome (PRRS) virus structural and non-structural proteins in virus pathogenesis. Anim. Health Res. Rev..

[5] Gao Z.Q., Guo X., Yang H.C. (2004). Genomic characterization of two Chinese isolates of porcine respiratory and reproductive syndrome virus. Arch. Virol..

[6] Tian K., Yu X., Zhao T., Feng Y., Cao Z., Wang C., Hu Y., Chen X., Hu D., Tian X. (2007). Emergence of fatal PRRSV variants: Unparalleled outbreaks of atypical PRRS in China and molecular dissection of the unique hallmark. PLoS ONE.

[7] Zhou L., Wang Z., Ding Y., Ge X., Guo X., Yang H. (2015). Nadc30-like strain of porcine reproductive and respiratory syndrome virus, China. Emerg. Infect. Dis..

[8] Xu H., Li C., Li W., Zhao J., Gong B., Sun Q., Tang Y.D., Xiang L., Leng C., Peng J. (2022). Novel characteristics of Chinese NADC34-like PRRSV during 2020-2021. Transbound. Emerg. Dis..

[9] Cheng T.Y., Campler M.R., Schroeder D.C., Yang M., Mor S.K., Ferreira J.B., Arruda A.G. (2022). Detection of Multiple Lineages of PRRSV in Breeding and Growing Swine Farms. Front. Vet. Sci..

[10] Van Geelen A.G.M., Anderson T.K., Lager K.M., Das P.B., Otis N.J., Montiel N.A., Miller L.C., Kulshreshtha V., Buckley A.C., Brockmeier S.L. (2018). Porcine reproductive and respiratory disease virus: Evolution and recombination yields distinct ORF5 RFLP 1-7-4 viruses with individual pathogenicity. Virology.

[11] Song S., Xu H., Zhao J., Leng C., Xiang L., Li C., Fu J., Tang Y.D., Peng J., Wang Q. (2020). Pathogenicity of NADC34-like PRRSV HLJDZD32-1901 isolated in China. Vet. Microbiol..

[12] Xie C.Z., Ha Z., Zhang H., Zhang Y., Xie Y.B., Zhang H., Nan F.L., Wang Z., Zhang P., Xu W. (2020). Pathogenicity of porcine reproductive and respiratory syndrome virus (ORF5 RFLP 1-7-4 viruses) in China. Transbound. Emerg. Dis..

[13] Yuan L., Zhu Z., Fan J., Liu P., Li Y., Li Q., Sun Z., Yu X., Lee H.S., Tian K. (2022). High Pathogenicity of a Chinese NADC34-like PRRSV on Pigs. Microbiol. Spectr..

[14] Yuan L., Zhu Z., Fan J., Li Q., Liu P., Li X. (2023). Efficacy of a commercial PRRSV vaccine on NADC34-like PRRSV challenge. Transbound. Emerg. Dis..

[15] Liu J., Liu C., Xu Y., Yang Y., Li J., Dai A., Huang C., Luo M., Wei C. (2022). Molecular Characteristics and Pathogenicity of a Novel Recombinant Porcine Reproductive and Respiratory Syndrome Virus Strain from NADC30-, NADC34-, and JXA1-Like Strains That Emerged in China. Microbiol. Spectr..

[16] Wang H., Feng W. (2024). Current Status of Porcine Reproductive and Respiratory Syndrome Vaccines. Vaccines.

[17] Chae C. (2021). Commercial PRRS Modified-Live Virus Vaccines. Vaccines.

[18] Renukaradhya G.J., Meng X.J., Calvert J.G., Roof M., Lager K.M. (2015). Live porcine reproductive and respiratory syndrome virus vaccines: Current status and future direction. Vaccine.

[19] Vu H.L.X., Pattnaik A.K., Osorio F.A. (2017). Strategies to broaden the cross-protective efficacy of vaccines against porcine reproductive and respiratory syndrome virus. Vet. Microbiol..

[20] Leng C., Zhang W., Zhang H., Kan Y., Yao L., Zhai H., Li M., Li Z., Liu C., An T. (2017). ORF1a of highly pathogenic PRRS attenuated vaccine virus plays a key role in neutralizing antibody induction in piglets and virus neutralization in vitro. Virol. J..

[21] Kappes M.A., Miller C.L., Faaberg K.S. (2015). Porcine reproductive and respiratory syndrome virus nonstructural protein 2 (nsp2) topology and selective isoform integration in artificial membranes. Virology.

[22] Tian D., Wei Z., Zevenhoven-Dobbe J.C., Liu R., Tong G., Snijder E.J., Yuan S. (2012). Arterivirus minor envelope proteins are a major determinant of viral tropism in cell culture. J. Virol..

[23] Xie J., Vereecke N., Theuns S., Oh D., Vanderheijden N., Trus I., Sauer J., Vyt P., Bonckaert C., Lalonde C. (2021). Comparison of Primary Virus Isolation in Pulmonary Alveolar Macrophages and Four Different Continuous Cell Lines for Type 1 and Type 2 Porcine Reproductive and Respiratory Syndrome Virus. Vaccines.

[24] Zhang H.L., Tang Y.D., Liu C.X., Xiang L.R., Zhang W.L., Leng C.L., Wang Q., An T.Q., Peng J.M., Tian Z.J. (2018). Adaptions of field PRRSVs in Marc-145 cells were determined by variations in the minor envelope proteins GP2a-GP3. Vet. Microbiol..

[25] Liu C., Zhang W., Gong W., Zhang D., She R., Xu B., Ning Y. (2015). Comparative Respiratory Pathogenicity and Dynamic Tissue Distribution of Chinese Highly Pathogenic Porcine Reproductive and Respiratory Syndrome Virus and its Attenuated Strain in Piglets. J. Comp. Pathol..

[26] Wang Y., Liang Y., Han J., Burkhart K.M., Vaughn E.M., Roof M.B., Faaberg K.S. (2008). Attenuation of porcine reproductive and respiratory syndrome virus strain MN184 using chimeric construction with vaccine sequence. Virology.

[27] Wang G., Yu Y., Zhang C., Tu Y., Tong J., Liu Y., Chang Y., Jiang C., Wang S., Zhou E.M. (2016). Immune responses to modified live virus vaccines developed from classical or highly pathogenic PRRSV following challenge with a highly pathogenic PRRSV strain. Dev. Comp. Immunol..

[28] Tian Z.J., An T.Q., Zhou Y.J., Peng J.M., Hu S.P., Wei T.C., Jiang Y.F., Xiao Y., Tong G.Z. (2009). An attenuated live vaccine based on highly pathogenic porcine reproductive and respiratory syndrome virus (HP-PRRSV) protects piglets against HP-PRRS. Vet. Microbiol..

[29] Xie J., Trus I., Oh D., Kvisgaard L.K., Rappe J.C.F., Ruggli N., Vanderheijden N., Larsen L.E., Lefèvre F., Nauwynck H.J. (2019). A Triple Amino Acid Substitution at Position 88/94/95 in Glycoprotein GP2a of Type 1 Porcine Reproductive and Respiratory Syndrome Virus (PRRSV1) Is Responsible for Adaptation to MARC-145 Cells. Viruses.

[30] Chen N., Li S., Li X., Ye M., Xiao Y., Yan X., Li X., Zhu J. (2020). The infectious cDNA clone of commercial HP-PRRS JXA1-R-attenuated vaccine can be a potential effective live vaccine vector. Transbound. Emerg. Dis..

[31] Yu P., Wei R., Dong W., Zhu Z., Zhang X., Chen Y., Liu X., Guo C. (2020). CD163^ΔSRCR5^ MARC-145 Cells Resist PRRSV-2 Infection via Inhibiting Virus Uncoating, Which Requires the Interaction of CD163 With Calpain 1. Front. Microbiol..

[32] Zhu Z., Zhang H., Zhang X., He S., Dong W., Wang X., Chen Y., Liu X., Guo C. (2020). Lipopolysaccharide Downregulates CD163 Expression to Inhibit PRRSV Infection via TLR4-NF-κB Pathway. Front. Microbiol..

[33] Zhu Z., Ye Z., Wang W., Li Y., Sun Z., Yu X., Tian K., Li X. (2024). A rescued virus from the infectious clone of a PRRSV NADC34-like strain exhibits high pathogenicity for nursery pigs. J. Integr. Agric..

[34] Zhu Z., Liu P., Yuan L., Lian Z., Hu D., Yao X., Li X. (2021). Induction of UPR Promotes Interferon Response to Inhibit PRRSV Replication via PKR and NF-κB Pathway. Front. Microbiol..

[35] Xu H., Li C., Gong B., Li W., Guo Z., Sun Q., Zhao J., Xiang L., Li J., Tang Y.D. (2023). Live-Attenuated Vaccine Derived from the SD-R Strain against NADC34-like Porcine Reproductive and Respiratory Syndrome Virus. Vaccines.

[36] Chen N., Ye M., Xiao Y., Li S., Huang Y., Li X., Tian K., Zhu J. (2019). Development of universal and quadruplex real-time RT-PCR assays for simultaneous detection and differentiation of porcine reproductive and respiratory syndrome viruses. Transbound. Emerg. Dis..

[37] Huang G., Liu X., Tang X., Du L., Feng W., Hu X., Zhu L., Li Q., Suo X. (2017). Increased Neutralizing Antibody Production and Interferon-γ Secretion in Response to Porcine Reproductive and Respiratory Syndrome Virus Immunization in Genetically Modified Pigs. Front. Immunol..

[38] Li C., Gong B., Sun Q., Xu H., Zhao J., Xiang L., Tang Y.D., Leng C., Li W., Guo Z. (2021). First Detection of NADC34-like PRRSV as a Main Epidemic Strain on a Large Farm in China. Pathogens.

[39] Sun Y.F., Liu Y., Yang J., Li W.Z., Yu X.X., Wang S.Y., Li L.A., Yu H. (2022). Recombination between NADC34-like and QYYZ-like strain of porcine reproductive and respiratory syndrome virus with high pathogenicity for piglets in China. Transbound. Emerg. Dis..

[40] Zhao H.Z., Wang F.X., Han X.Y., Guo H., Liu C.Y., Hou L.N., Wang Y.X., Zheng H., Wang L., Wen Y.J. (2022). Recent advances in the study of NADC34-like porcine reproductive and respiratory syndrome virus in China. Front. Microbiol..

[41] Kwon B., Ansari I.H., Pattnaik A.K., Osorio F.A. (2008). Identification of virulence determinants of porcine reproductive and respiratory syndrome virus through construction of chimeric clones. Virology.

[42] Zhao K., Gao J.C., Xiong J.Y., Guo J.C., Yang Y.B., Jiang C.G., Tang Y.D., Tian Z.J., Cai X.H., Tong G.Z. (2018). Two Residues in NSP9 Contribute to the Enhanced Replication and Pathogenicity of Highly Pathogenic Porcine Reproductive and Respiratory Syndrome Virus. J. Virol..

[43] Ellingson J.S., Wang Y., Layton S., Ciacci-Zanella J., Roof M.B., Faaberg K.S. (2010). Vaccine efficacy of porcine reproductive and respiratory syndrome virus chimeras. Vaccine.

[44] Loving C.L., Osorio F.A., Murtaugh M.P., Zuckermann F.A. (2015). Innate and adaptive immunity against Porcine Reproductive and Respiratory Syndrome Virus. Vet. Immunol. Immunopathol..

[45] Kappes M.A., Miller C.L., Faaberg K.S. (2013). Highly divergent strains of porcine reproductive and respiratory syndrome virus incorporate multiple isoforms of nonstructural protein 2 into virions. J. Virol..

