# Supplementary figures and images for: A Live-Attenuated Chimeric Vaccine Candidate Against the Emerging NADC34-Like PRRSV
Source: Vet Sci. 2025 Mar 19;12(3):290. doi: 10.3390/vetsci12030290 (PMC11946239; doi:10.3390/vetsci12030290)

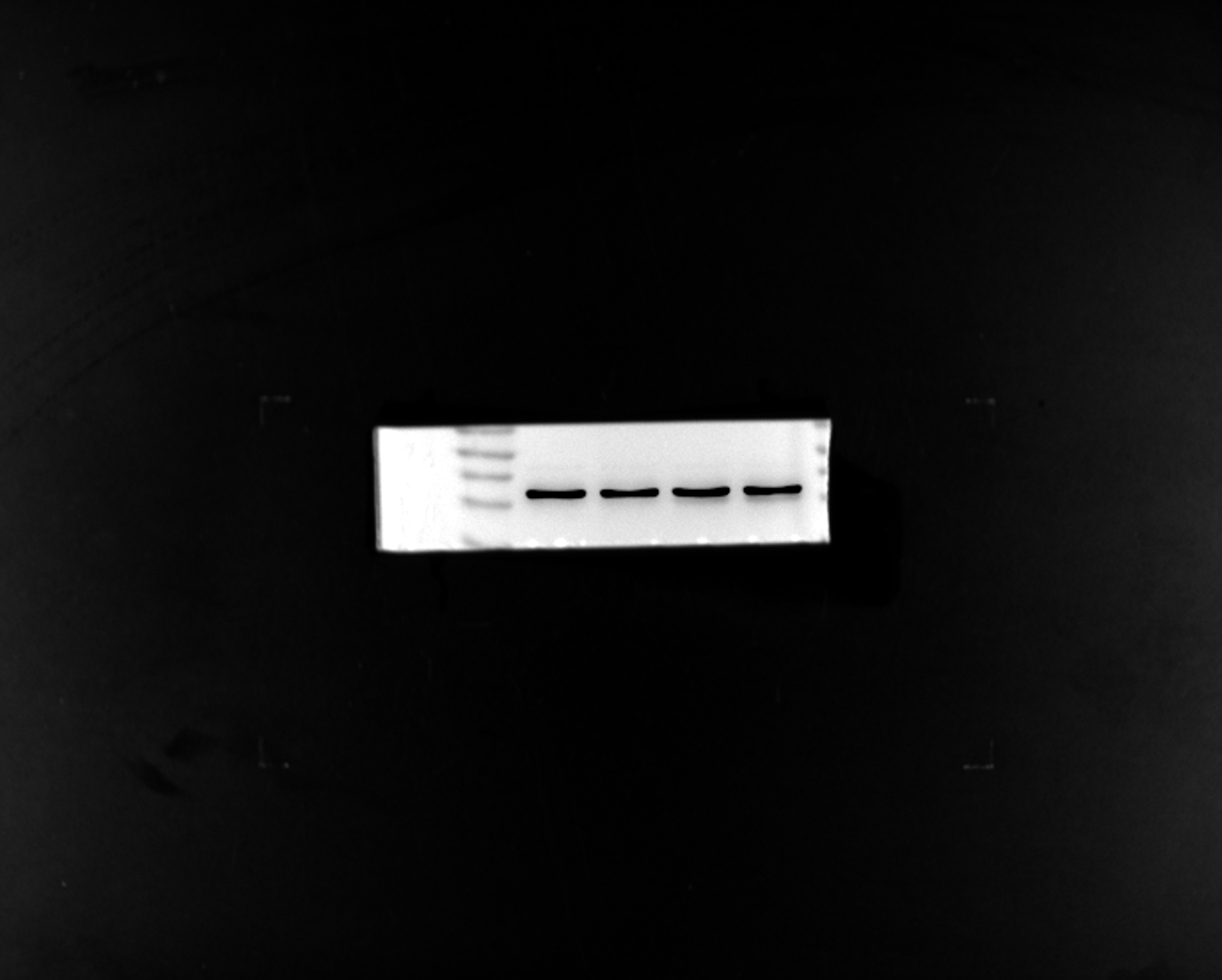

Supplement: Supplementary file 1 [file vetsci-12-00290-s001.zip › Fig 2B GAPDH.Tif]

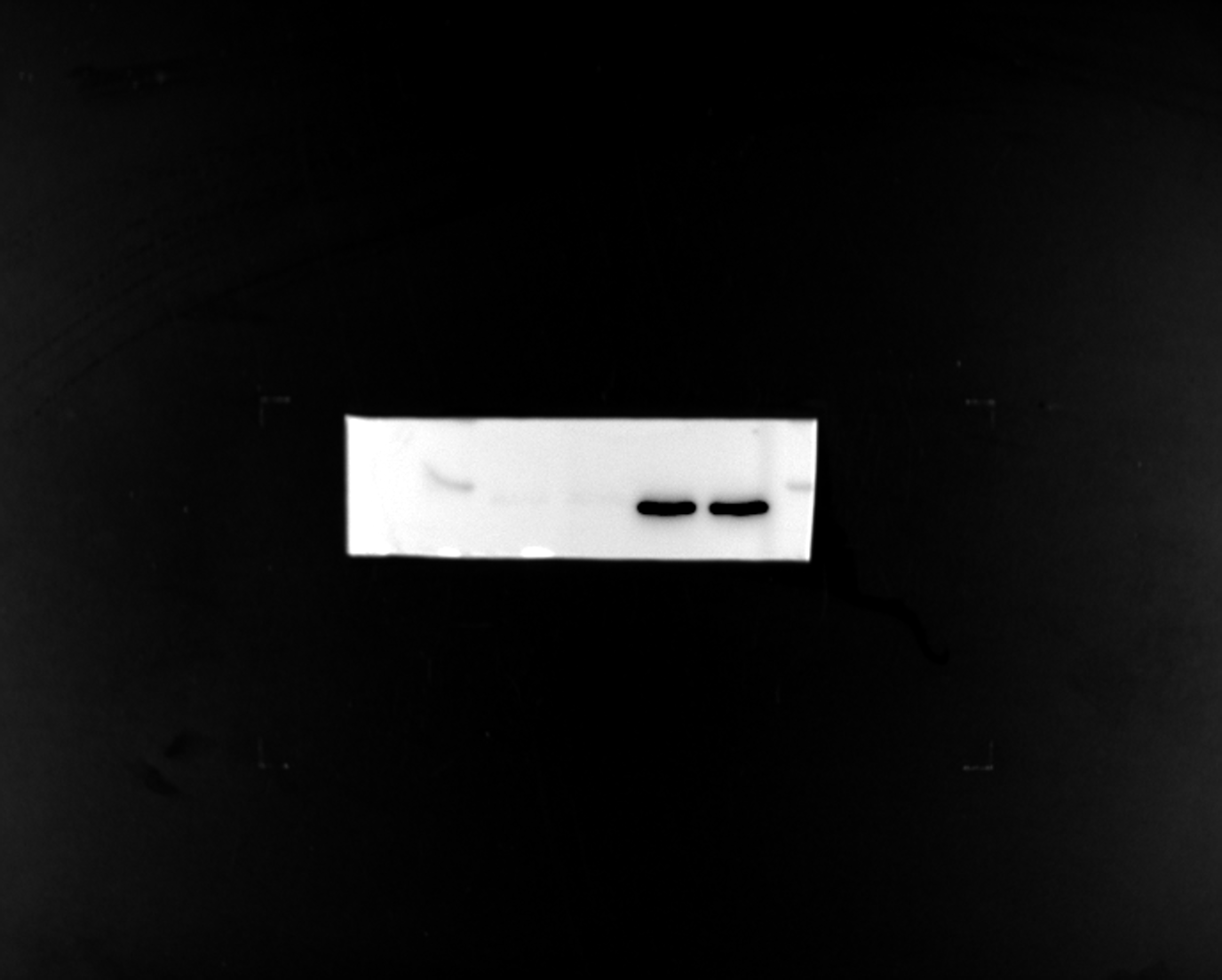

Supplement: Supplementary file 1 [file vetsci-12-00290-s001.zip › Fig 2B PRRSV N.Tif]
